# Evaluation of Galdakao-modified Valdivia position in endoscopic management of malignant ureteric obstruction

**DOI:** 10.1007/s11255-022-03109-4

**Published:** 2022-01-27

**Authors:** Ahmed Emam, Mohamed Elmoazen, Mohamed Shabayek, Amr M. Zriek, Hany Hamed Gad

**Affiliations:** 1 Department of Urology, Ain Shams University, 38 Abbasia, Cairo, 11591 Egypt; 2 Department of Urology, Ahmed Maher Teaching Hospital, Cairo, Egypt

**Keywords:** Galdakao-modified Valdivia position, Ureteric obstruction, Malignant ureteric obstruction, Ureteric stenting, Antegrade ureteric stenting

## Abstract

**Background:**

Malignant ureteric obstruction (MUO) due to pelvic malignancies is challenging for endourological management and carries high failure rates for retrograde cystoscopic ureteric stenting.

**Methods:**

We adopted Galdakao-modified Valdivia (GMV) position in the management of MUO in an operating room equipped with a C-arm fluoroscopy unit and an ultrasound device. We prospectively studied the added value of this approach in 50 cases who failed retrograde ureteric stenting.

**Results:**

Thirty-seven (74%) cases were done under a high level of spinal anesthesia. Mean operative time was 62 min. Antegrade ureteric stenting succeeded in 45/50 (90%) patients who failed retrograde ureteric stenting. GMV position facilitated simultaneous retrograde and antegrade management of MUO. Eight patients (16%) underwent auxiliary cystoscopic procedures to reduce the mass over the ureteric orifice (UO) guided by antegrade methylene blue or over a probing antegrade guidewire. Nephrostomy tube was inserted in the same setting in 16/50 (32%) cases. Antegrade flow of contrast to the bladder (*P* < 0.001) and ureteric kinks rather than tight stenosis or infiltration of UO (*P* = 0.014) were significantly associated with the success of antegrade ureteric stenting. No major complications were encountered.

**Conclusion:**

GMV position is an ideal choice for management of MUO as it allows simultaneous access to the lower and the upper urinary systems to accomplish ureteric stenting either in a retrograde or an antegrade fashion as well as the ability to insert a nephrostomy tube in the same setting, thus shortening the inpatient care and this should be the standard of care in cases with MUO.

## Introduction

Hydronephrosis results from urinary tract obstruction that can be intrinsic or extrinsic and can result from both benign and malignant etiologies. Extrinsic obstruction is caused by compression or mural infiltration of the ureteric wall by a surrounding mass or urologic, gynecologic, or colorectal tumor [1].

External urinary tract drainage with a percutaneous nephrostomy catheter (PCN), carries a high incidence of complications in the long-term management, such as infection and dislocation [2]. Internal ureteric stents avoids PCN associated problems and avoid the use of an external nephrostomy bag thus provides better quality of life [2]. However, retrograde ureteric stenting can be difficult or even impossible in patients with MUO [3]. It may fail in up to 50% in patients with distal and extra-ureteral obstruction caused by malignancies [4]. In such cases, the only option left is the insertion of a PCN with or without an attempt of antegrade ureteric stenting [1].

The GMV position allows for simultaneous retrograde and antegrade urinary tract approaches without patient repositioning [5].

## Patients and methods

### Patients

After obtaining approval from the ethical committee at Ain shams University (No. 344/2016), 50 patients with MUO whether urological or non-urological causes who failed retrograde ureteric stenting were included in this prospective study from 2016 until 2020, at the Department of Urology, Ain Shams University, Cairo, Egypt.

All patients had informed consent, full laboratory investigations including renal function tests, preoperative anesthesiological evaluation, an antegrade nephrostogram if there was a preoperatively inserted PCN, an intravenous urography (IVU) study if not contraindicated or an intraoperative pyelogram to detect the level and length of the obstructed ureteric segment, the flow of dye distal to obstruction to the bladder and the presence of ureteric kinks. The findings were evaluated as predictors of the success or failure of the procedure.

### Technique

**Fig. 1:**
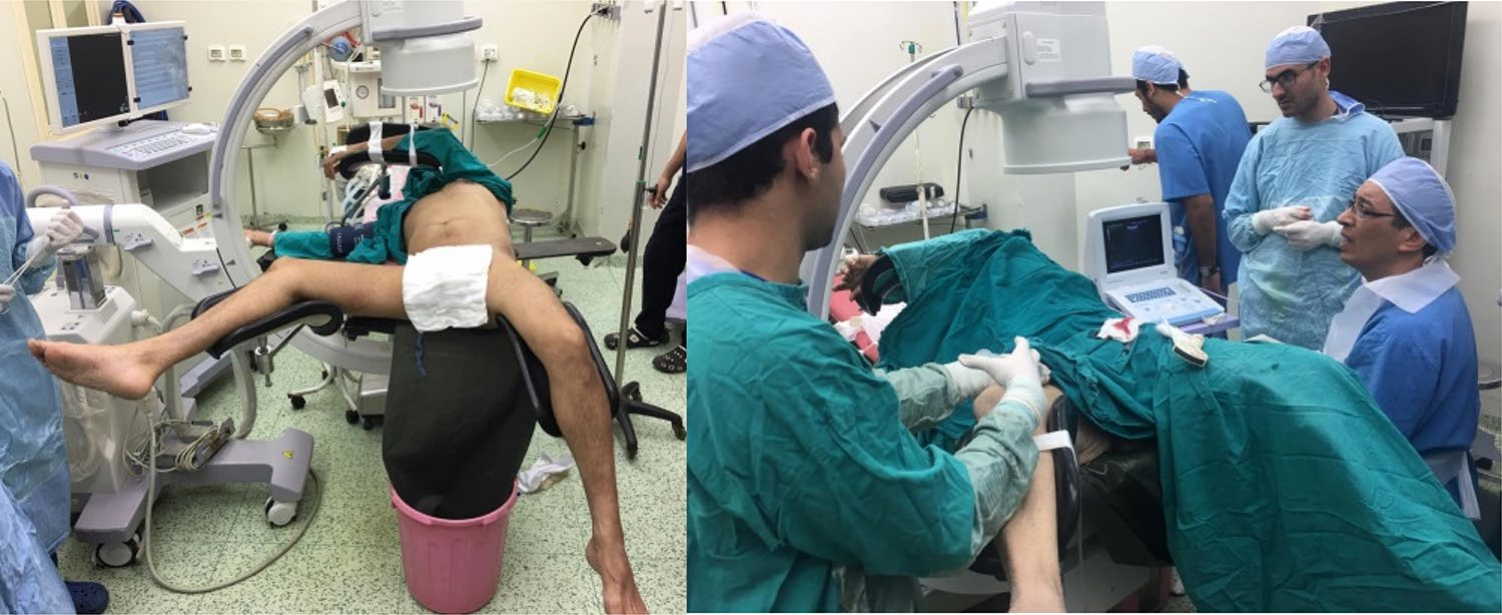
Patient positioning (GMV position) and operative room setup

Cases were done under high-level spinal anesthesia in 37/50 (74%) patients and under general anesthesia in 13/50 (26%) patients. Patients were positioned in GMV position (ipsilateral lower leg in extension and the other in hip flexion) (Fig. 1) in an operating room equipped with a mobile C-arm fluoroscopy unit and a beside ultrasound device (3.5 MHz transducer) and received preoperative antibiotic prophylaxis.

In all cases, we started with a diagnostic cystoscopy, retrograde pyelography, and a retrograde stenting attempt to identify possible causes of retrograde stenting failure and exclude surgeon or instrumental factors.

The antegrade ureteric stenting procedure started by an ultrasonography-guided puncture preferentially through a mid or upper pole calyx as it provides a more favorable angle to the ureter than the lower calyx. This was followed by an antegrade pyelogram and passing a hydrophilic 0.035-inch guidewire (Terumo guidewire) as a working guidewire down the ureter into the bladder. Another guidewire was left during the whole procedure as a safety guidewire.

**Fig. 2:**
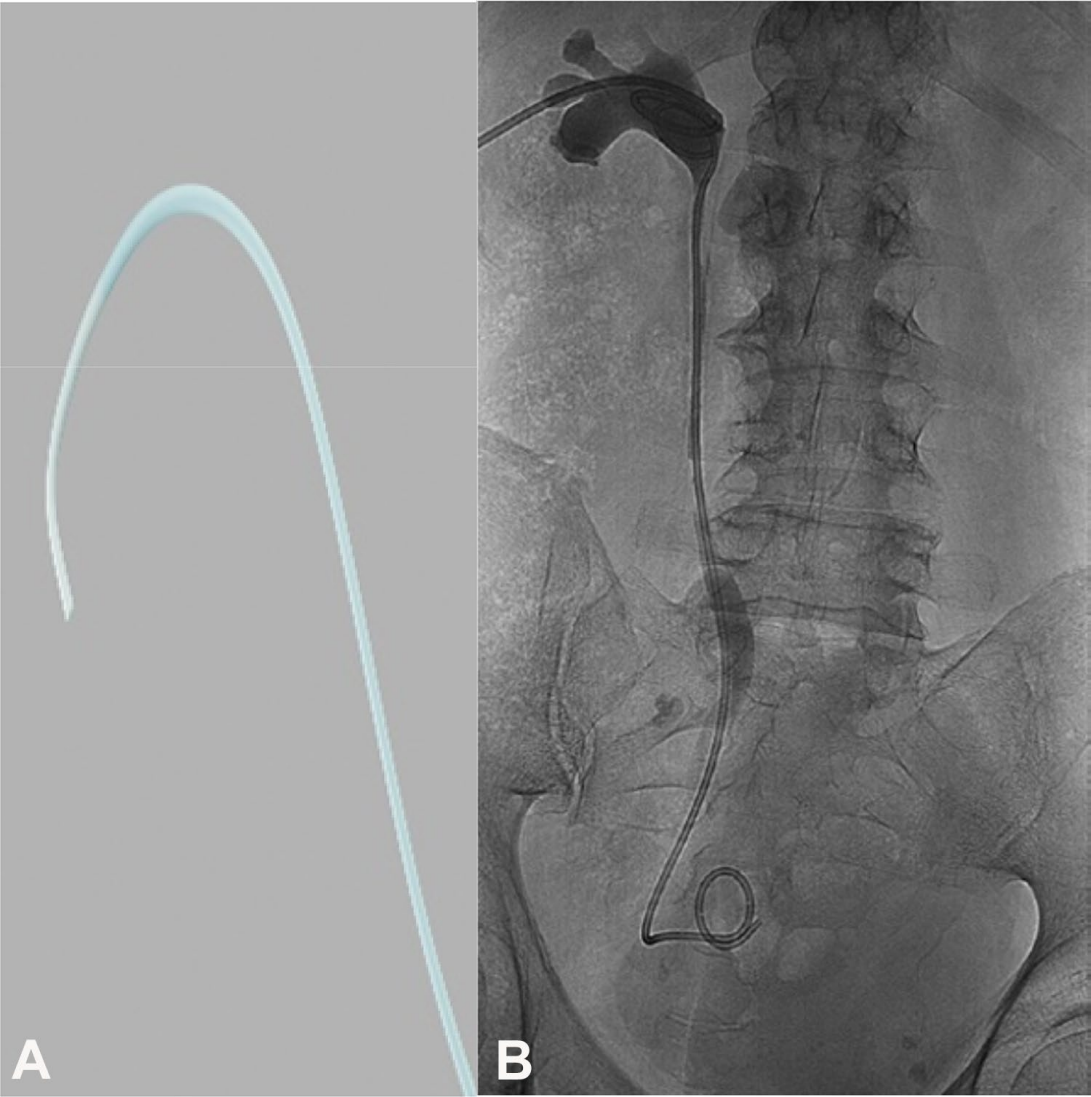
**A** Multipurpose angiographic (MPA) catheter: cobra head catheter (Cook Medical Inc.). **B** Antegrade ureteric stent and nephrostomy tube passed effectively at the end of the procedure

A curved tip multipurpose angiography catheter (MPA) (Cook Medical Inc.) was used to manipulate obstacles that caused the retrograde access to fail (Fig. 2a). MPA allowed the direction of the guidewire through the stricture followed by guidewire retrieval from the bladder and ureteric stenting (Fig. 2b). Long-term 7 Fr Coloplast ureteral stents were used.

Maneuvers used during antegrade ureteric stenting were as follows: If resistance was encountered because of ureteric kinks, an antegrade catheter was advanced to the level of the kink, and dilute contrast was injected to outline the ureteral path. MPA catheter was used in conjunction with a floppy tip guidewire to manipulate the kink. This was also aided with the change in the patient's respiratory effort or slight upward push to the kidney or slight upward traction on an antegrade balloon dilator inflated above the kink to alter the degree of angulation enough to overcome the kinks and achieve a successful catheterization.

Resistance secondary to ureteric stricture was manipulated with a catheter introduced to the level of the stricture and dilute contrast was injected to map the ureteral course. MPA catheter was positioned about 1 cm above the obstacle and a floppy tip guidewire is inserted. Rotation of the catheter while gently probing allowed the guidewire to approach the stenosis at multiple angles until the opening was located.

In face of bladder mass or prostatic cancer occluding and obscuring the UO, reduction of the mass was done through trans-urethral resection of the bladder tumor (TURBT) or the prostate (TURP) guided by antegrade methylene blue dye or over a probing antegrade guidewire. Once the guidewire was retrieved out of UO, through and through railroad access (body floss technique) was obtained. Tension was maintained on both ends of the guidewire and an antegrade ureteric stent was placed. A covering nephrostomy was left for 2 days at the end of the procedure depending on the intraoperative complications (extravasation or bleeding).

### Statistics

Success rates were recorded and plotted against the parameters of preoperative nephrostogram, IVU and/or intraoperative pyelogram to identify the independent predictors of the success of ureteric stenting. Operative time, hospital stay, intraoperative and early postoperative complications (1 week after discharge) were recorded.

## Results

Fifty patients with MUO were enrolled in this prospective study. Patient characteristics are summarized in Table 1.

**Table 1:** Patient characteristics

	(*N* = 50)
Age (years)	
Mean ± SD	54.400 ± 7.284
Range	22–66
Sex	
Male	29(58%)
Female	21(42%)
Body mass index (BMI) (Kg/m^2^)	
Mean	28.840 ± 3.893
Range	20–40
Primary cancer causing MUO	
Bladder	20 (40%)
Prostate	10 (20%)
Cervix	7 (14%)
Endometrium	6 (12%)
Colon	7 (14%)
Level of obstruction	
Distal ureter	37 (74%)
Mid ureter	13 (26%)
Degree of hydronephrosis	
Moderate	22 (44%)
Severe	28 (56%)
Pre-operative serum creatinine (mg/dL)	
Mean ± SD	2.182 ± 0.582
Range	1.3–3.7
Post-operative serum creatinine (mg/dL)	
Mean ± SD	1.946 ± 0.648
Range	1–3.5

Out of 20 patients with urothelial bladder cancer, 15 patients had muscle-invasive bladder cancer and 5 patients had non-muscle-invasive bladder cancer. In the non-muscle invasive bladder cancer, the obstruction was due to the bladder mass encroaching the UO in three cases and delayed UO stenosis as a consequence of a prior TURBT in two cases.

Causes of failure of retrograde ureteric stenting were the inability to identify the UO in 30/50 (60%) of patients (all of whom with bladder or prostatic carcinomas) and tight ureteric stenosis or kinks in 20/50 (40%) patients.

Antegrade ureteric stenting succeeded in 45/50 (90%) patients and failed in 5/50 (10%) patients, among whom a case of endometrial cancer had a tight ureteric stenosis that could not be negotiated and four cases (two cases with bladder cancer and two cases with prostate cancer) in which UO was not identified despite the use of antegrade methylene blue or a probing antegrade guidewire. It was our protocol not to resect over a presumed site of UO unless it was guided by antegrade methylene blue or a probing guidewire.

GMV position facilitated simultaneous auxiliary cystoscopic procedures in eight (16%) patients (TURBT in six patients and TURP in two patients) to reduce the mass over the UO guided by antegrade methylene blue or antegrade guidewire probing. Nephrostomy tube was inserted in the same setting in 16/50 (32%) patients including 5/50 patients who failed both retrograde and antegrade ureteric stenting and 11/50 patients to help of recovery from complications (e.g., bleeding or extravasation).

Antegrade flow of contrast to the bladder (*P* < 0.001) and ureteric kinks rather than tight stenosis or infiltration of UO (*P* = 0.014) were significantly associated with the success of antegrade ureteric stenting (Table 2).

**Table 2:** Correlation of the dye findings either though IVU or antegrade pyelogram to the success or failure of the antegrade ureteric stenting

	Antegrade ureteric stenting			*P* value
	Total (50)	Success (34)	Fail (16)	
	*N* (%)	*N* (%)	*N* (%)	
Antegrade flow of dye to the bladder				
Yes	28 (56)	27 (96.4)	1(3.6)	< 0.001
No	22 (44)	7 (31.8)	15 (68.2)	
Presence/absence of ureteric kinks				
Kink	37 (74)	29 (78.4)	8 (21.6)	0.014
No	13 (26)	5 (38.5)	8 (61.5)	
Length of obstructed segment				
< 1 cm	34 (68)	26 (76.5)	8 (23.5)	0.103
> 1 cm	16 (32)	8 (50)	8 (50)	

Mean operative time was 62 ± 26 min (range 30–120 min) and mean hospital stay was 1.8 ± 0.7 days (range 1–3 days). No complications were encountered in 16/50 (32%) of patients, while 27/50 (54%) had mild hematuria (the mean drop of hemoglobin did not exceed 1 g/dL and there was no need for blood transfusion) and 7/50 (14%) had contrast extravasation yet did not affect the procedure. All the complications subsided with conservative measures.

## Discussion

MUO is a frequent complication of advanced pelvic or retroperitoneal malignancy [6].

In clinical practice, ureteral stents are commonly used to circumvent obstructions, and retrograde ureteric stenting is preferable to PCN. However, retrograde stenting failure for extrinsic ureteral obstruction was seen in 42% of ureteral units [7], compared to 9% in cases of intrinsic ureteral obstruction [8]. Cancer diagnosis, baseline creatinine > 1.3 mg/dL, and post-stent systemic treatment were predictors of failure of retrograde ureteral stenting [7]. In patients with MUO, retrograde stenting failure ranged from 21 to 52% in prior studies [8–11]. In such patients, gastrointestinal malignancy, poor performance status, and severe hydronephrosis were independent predictors of retrograde stent failure [10]. Stent failure was also predicted by the location of the ureteral obstruction, with stents placed for distal or middle ureteral obstructions being more likely to fail [12]. The majority of retrograde stenting failures in our study, as well as other studies [8, 11], were due to an inability to identify UO. Patients with MUO caused by a gynecologic malignancy, on the other hand, had a better prognosis than those with other malignancies [9].

In cases of MUO, antegrade ureteric stenting had a higher success rate than retrograde ureteric stenting. Chitale and colleagues recommended two-stage antegrade ureteric stenting over endoscopic retrograde ureteric stenting in the management of MUO, reporting a success rate of 21% for endoscopic retrograde ureteric stenting versus 98% for two-stage antegrade ureteric stenting in 65 patients with MUO, 24 of whom had failed an attempt at endoscopic retrograde ureteric stenting [13]. Similarly, Uthappa and colleagues attempted retrograde stenting in 50 ureters in 30 MUO patients and observed a 50% success rate (*n* = 25/50). Antegrade stenting was successful in 96% of individuals who failed retrograde stenting (*n* = 24/25 ureters) [11]. Harding also reported a 92% (34/37 ureters) success rate for antegrade ureteric stenting in 25 MUO patients for whom retrograde ureteric stenting was unfeasible [14].

Inserting a nephrostomy tube with one or two-stage antegrade ureteric stenting by interventional radiology (IR) in a separate setting is the current approach for patients who fail a retrograde ureteric stenting attempt. We adopted GMV position in an operating room equipped with a fluoroscopy unit and an ultrasound device as an ideal setup giving those fragile MUO patients the best chance of stenting success by combining the privileges of both retrograde and antegrade accesses in the same setting while under anesthesia, avoiding a secondary procedure, reducing inpatient care and hospital stay. We were able to achieve a stenting success rate of 90% in MUO patients who failed retrograde ureteric stenting. Patients with malignancy obscuring UO benefited the most from this approach. Antegrade flow of contrast to the bladder and ureteric kinks rather than tight obstruction or infiltration of UO were significantly associated with the success of antegrade ureteric stenting.

In another study, causes of antegrade ureteric stenting failure included poor angulation of the percutaneous tract, tortuous dilated ureters, tight obstruction, wedging of stent assembly components due to high resistance, and difficulty in the positioning of the proximal pigtail [15].

In our study, mild complications were encountered in 34/50 (64%) of patients; 27/50 (54%) had mild hematuria and 7/50 (14%) had contrast extravasation. All the complications subsided with conservative measures. Rao and colleagues performed a retrospective review of 165 antegrade ureteric stent insertions. They reported 3% (5/165) patients, with silent ureteric perforation and an extra-anatomic placement of ureteric stent with pelvic urinoma and delayed retroperitoneal abscesses [16]. Borell and colleagues reported a case of peri-renal hematoma among 27 antegrade ureteric stent insertions [17].

## Conclusions

Based on our experience, GMV position in an operating room equipped with a C-arm fluoroscopy unit and an ultrasound device is an ideal setup for managing MUO especially with malignancy obscuring UO as it allows simultaneous access to the lower and the upper urinary systems to accomplish ureteric stenting either retrogradely or antegradely, as well as well as the ability to insert a nephrostomy tube in the same setting while under anesthesia, avoiding a secondary procedure, and reducing inpatient care or hospital stay and this should be the standard of care in MUO cases. Pre-operative and intraoperative contrast studies can be used to predict the success of the procedure.

We recommend that at least one team in each urology department be familiar with this approach, or that the case be double booked with an interventional radiologist who can join in the event of retrograde stenting failure. This will ensure the best possible patient care.

## Abbreviations

GMV: Galdakao-modified Valdivia
MPA catheter: Multipurpose angiography catheter
MUO: Malignant ureteric obstruction
PCN: Percutaneous nephrostomy
TURBT: Trans-urethral resection of the bladder tumor
TURP: Trans-urethral resection of the prostate
UO: Ureteric orifice
